# Plasmon-actuated nano-assembled microshells

**DOI:** 10.1038/s41598-017-17691-6

**Published:** 2017-12-19

**Authors:** Makiko T. Quint, Som Sarang, David A. Quint, Amir Keshavarz, Benjamin J. Stokes, Anand Bala Subramaniam, Kerwyn Casey Huang, Ajay Gopinathan, Linda S. Hirst, Sayantani Ghosh

**Affiliations:** 1 School of Natural Sciences, University of California, Merced, CA 95344 USA; 2 Department of Bioengineering, Stanford University, Stanford, CA 94305 USA; 3 Department of Microbiology and Immunology, Stanford University School of Medicine, Stanford, CA 94305 USA; 4 School of Engineering, University of California, Merced, CA 95344 USA

## Abstract

We present three-dimensional microshells formed by self-assembly of densely-packed 5 nm gold nanoparticles (AuNPs). Surface functionalization of the AuNPs with custom-designed mesogenic molecules drives the formation of a stable and rigid shell wall, and these unique structures allow encapsulation of cargo that can be contained, virtually leakage-free, over several months. Further, by leveraging the plasmonic response of AuNPs, we can rupture the microshells using optical excitation with ultralow power (<2 mW), controllably and rapidly releasing the encapsulated contents in less than 5 s. The optimal AuNP packing in the wall, moderated by the custom ligands and verified using small angle x-ray spectroscopy, allows us to calculate the heat released in this process, and to simulate the temperature increase originating from the photothermal heating, with great accuracy. Atypically, we find the local heating does not cause a rise of more than 50 °C, which addresses a major shortcoming in plasmon actuated cargo delivery systems. This combination of spectral selectivity, low power requirements, low heat production, and fast release times, along with the versatility in terms of identity of the enclosed cargo, makes these hierarchical microshells suitable for wide-ranging applications, including biological ones.

## Introduction

Research involving nanomaterials has brought to light their intriguing electronic, magnetic and optical properties, so different from their bulk counterparts^1–5^. Amongst these, metallic nanoparticles are of particular interest, given their ability to modulate light-matter interactions by manipulating electromagnetic fields through localized surface plasmon resonances (LSPR)^6^. Plasmonic phenomena have found use in numerous applications, including biochemical sensing^7,8^, biomedical diagnostics and therapy^9–11^, high-resolution optical imaging^12,13^, optical switching^14^, photovoltaic devices^15,16^, energy storage^17,18^, and photocatalysis^19^. Gold nanoparticles (AuNPs) have LSPR in the visible to near infrared spectral region and is therefore the most popular candidate in typical applications, though silver, aluminum and platinum are studied as well^20–22^. In order to fully exploit plasmonics and reliably design devices with consistent performance, research efforts have been focusing on developing ordered assemblies of plasmonic nanosystems. These include both top-down and bottom-up approaches, but the latter has greater advantages in terms of versatility of geometry, architecture and functionality. Assemblies of colloidal AuNPs that have been achieved range from simple dimers and trimers^23^ to nanoclusters^24^, from one dimensional chains^25^ to networks^26^. Recently, novel arrangements such as core-satellite structures have also been assembled^27,28^, and these offer the added convenience of heterogenous formations. The process of assembly is directed via linkers with which AuNPs are functionalized, and those that have demonstrated the most success include DNA molecules^29^, organic ligands^30^ and linear^31^ and branched polymers^23^.

Moving beyond assembly, the next capability that would greatly enhance the functionality of these assemblies is responsiveness to external stimuli. Tuning the spectral position of the LSPR *in situ* is one approach, usually achieved by modulating the dielectric constant of the medium surrounding the AuNPs^32,33^. The other is utilizing the photothermal effect, where resonant optical excitation is absorbed by the AuNPs, which heats the crystal lattice, in turn transferring the heat to the surrounding medium. This phenomenon has potential application in targeted drug delivery, and such systems with release mechanisms that can be remotely triggered using stimuli such as electric and magnetic fields, as well as optical excitation, have been the subject of extensive research^34–41^. In case of optical actuation, the power, wavelength, and duration of incident light required is strongly dependent on the components used for construction of the capsule. And while it is critical for the capsules to provide robust leakage-free containment, the excitation power used to trigger release must be below the American National Standards Institute (ANSI) maximum permissible exposure limit. For visible excitation at ~500 nm, this limit is 2.5 mW for up to 10 min of illumination^42^. Optimizing these parameters simultaneously is challenging. One approach using silica-gold core-shell composite NPs for encapsulation and light-activated release utilizes 15–100 mW of illumination over tens of minutes^34^. AuNP-assembled nano-micelles and cross-linked nano-vesicles can release contents in under 10 min of excitation, but require higher powers (100–250 mW)^35^. With few exceptions^36^, composite structures and complexes that combine AuNPs with soft materials such as polymers, gels, and liposomes have lower power requirements (10–65 mW) at the cost of longer release times (10–40 min)^37^. Other methods using AuNP-DNA complexes^38^ or rare-earth NPs require power on the order of 1 W^39^.

**Figure 1:**
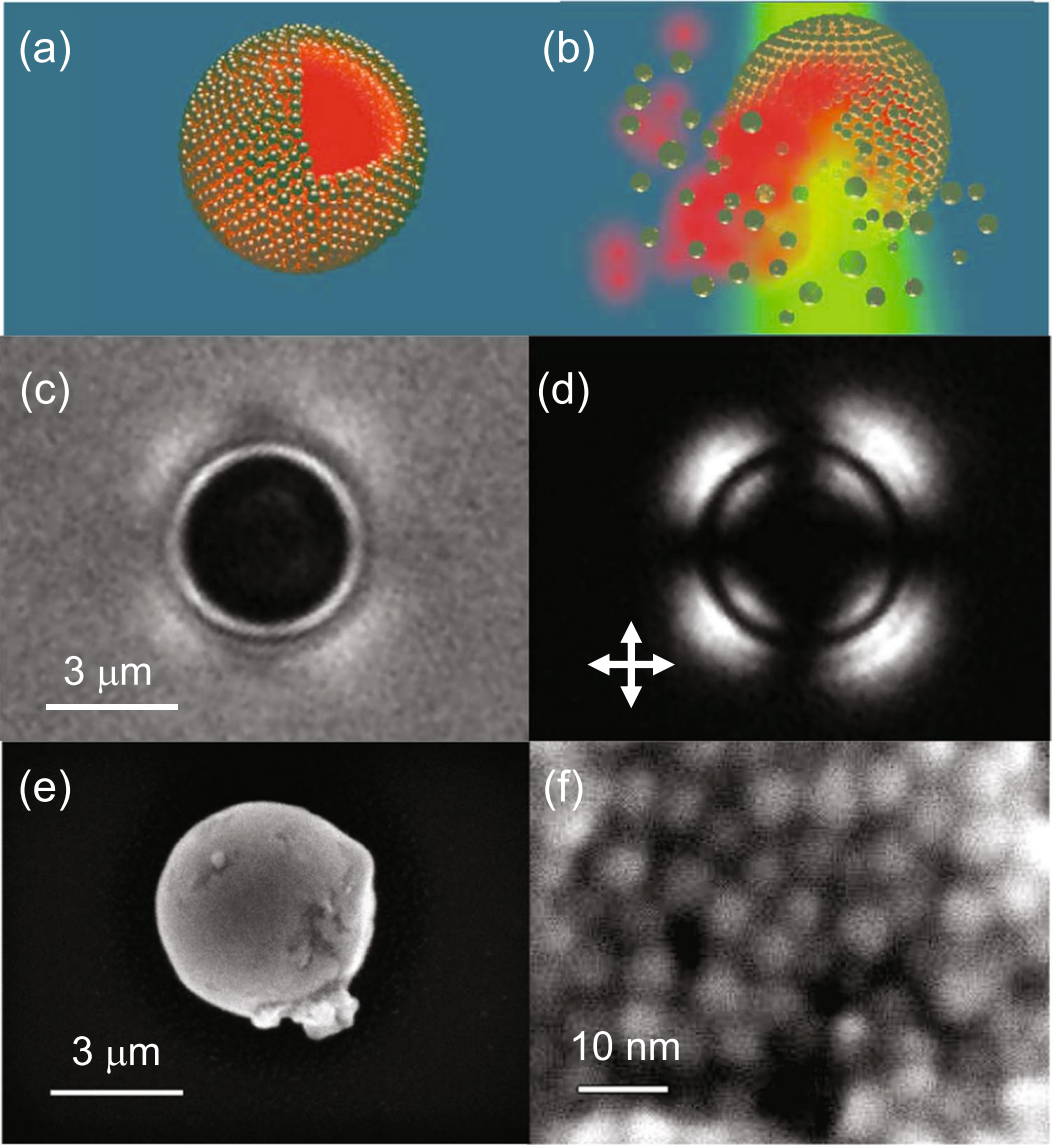
Characterization of a nano-assembled microshell (NAM). Schematic of (NAM) (**a**) An intact structure with sectional cut-out shows the encapsulated dye within. The wall is multiple layers thick. (**b**) Illumination by green light, resonant with the localized surface plasmon resonance (LSPR) of the nanoparticles in the wall, disrupts the structure due to photothermal heating, releasing the contents within. (**c**) Bright-field and (**d**) cross-polarized images of a NAM in liquid crystal medium. (**d**) SEM image of a NAM extracted from suspension and placed on an indium tin oxide coated glass slide. (**e**) Close-up of the same structure in (**d**), showing individual AuNPs that form the wall.

We present a liquid crystal (LC) phase-templated mechanism using AuNPs functionalized with mesogenic (LC-like) ligands (Figure S1)^43,44^ that produces nano-assembled microshells (NAMs) 1–5 µm in diameter with closely spaced nanoparticles constituting the shell wall, schematically represented in Fig. 1a. Using plasmon actuation via shining light of wavelength close to the AuNP LSPR, we show that cargo encapsulated within the NAM can be released in a spatially controlled manner, where localized heating breaks the shell wall via the photothermal effect. In contrast to most other techniques, our mechanism does not require the fabrication of multicomponent capsules, simplifying the synthesis and cargo encapsulation processes. Moreover, the rigidity of the shell wall ensures leakage-free containment over months. The NAMs were robust to leakage even when the shells were extracted from the native host and re-suspended in other solvents. Our NAMs are activated using <2 mW of incident power, and full release is obtained within 1–5 s (Fig. 1b). These data are by far the most optimized combination of power requirement and release time reported, and is made possible by the ideal packing of AuNPs achieved in the shell wall. The inter particle spacing, controlled by the length of ligands, allows the walls to have a high enough density of particles to allow low power requirements, and therefore rapid release times; but the spacing is larger than what is needed for strong inter particle coupling, which in turn permits an accurate estimate of the temperature increase due to the photothermal effect. Our calculations are verified by comparison with experimental quantification of bubble expansion rates, and indicate a most moderate rise of 50 °C which is a significant step toward the realization of photothermally activated capsules, with a broad range of applications in cargo encapsulation and delivery protocols.

## Results and Discussion

### Imaging-based characterization

Careful choice of mesogenic ligand-functionalized AuNPs dispersed in an anisotropic host phase (such as an LC) provides an excellent route toward directed assembly in our approach. A rigid mesogenic segment built into a long-chain hydrocarbon molecule can align with the surrounding host phase, decreasing the free-energy cost of dispersing AuNPs in the host (Figure S1). The ligand synthesis and AuNP functionalization was based on our prior work^43,44^. The host phase in this case is the nematic liquid crystalline (NLC) phase, where the LC molecules possess orientational order labeled by the director axis, but not long-range positional order. We monitored shell formation as the process occurred using bright-field microscopy. Figure 1c is a snapshot of one NAM taken as the shell formation is complete. Shell morphology and structure were characterized using polarized optical microscopy (POM) and scanning electron microscopy (SEM).

The inherent anisotropy of LC molecules makes them birefringent, and under POM, the relative orientation of the director axis with respect to the crossed-polarizers in the microscope determine the intensity of the transmission images. In our case, the director, was aligned perpendicular to the plane of the sample, which is known as homeotropic alignment, which should make the NLC film appear dark under POM. In Fig. 1d, this is the case away from the NAM. Closer to the shell wall however, the POM images showed a radially contrasting pattern. When spherical objects such as these are dispersed in a homeotropically aligned NLC, their surfaces induce director distortions^45^, and adjacent LC molecules acquire a different orientation compared with the director of the bulk of the film. The NAMs presented in this work exhibit planar surface anchoring as reported in our previous study^43^, with local LC molecules orienting parallel to their spherical surface. As a result, the birefringence image in Fig. 1d exhibits the characteristic cross pattern between crossed polarizers and is a confirmation of the three-dimensionality of the shells.

Using SEM, we observed shells post-extraction from the host NLC (Fig. 1e), which leads to slight deformation. Higher resolution SEM showed a densely-packed arrangement of the individual AuNPs in the shell wall (Fig. 1f), and small angle X-ray scattering (SAXS) indicated that the mean inter-particle separation of the AuNPs in the wall is 12.1 nm (Figure S2). This configuration turns out to be optimal for our purposes as this high AuNP density is critical to allow rapid actuation via low power.

### High containment capability

**Figure 2:**
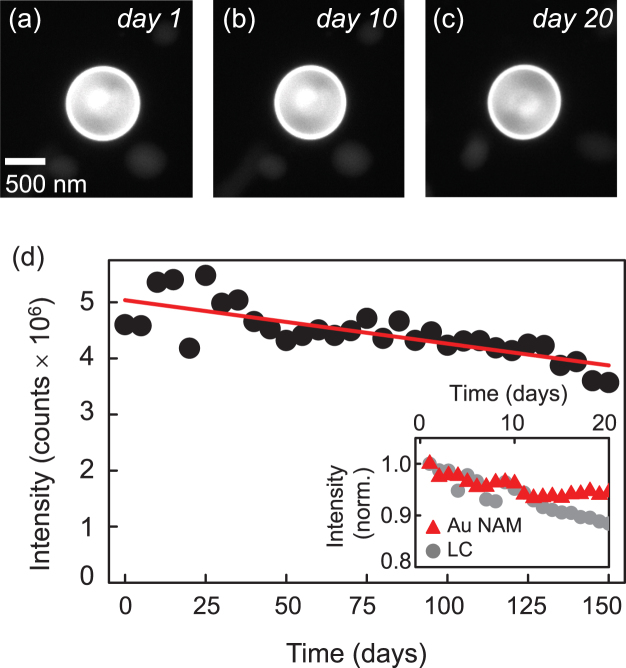
NAMs exhibit little to no leakage over many months. (**a**–**c**) Fluorescence images of Lumogen F Red encapsulated in a NAM. (**d**) Dye intensity measured over five months. Inset: dye intensity encapsulated in shells compared to that of dye suspended in liquid crystal alone. The quantitative agreement between the two over the first ~10 days indicates that the small decrease is likely due to photo bleaching.

For encapsulation within the NAMs, we used Lumogen F Red 300 (BASF), a dye with a high quantum yield. We dissolved the dye in toluene and added the dye to the NLC-AuNP mixture. Following sonication and shell formation, we spun down the NAMs with a centrifuge (8000 rpm for 30 min), discarded the supernatant, and re-suspended the dye-containing NAMs in dye-free NLC. We repeated the process five times until we achieved a clear contrast between the shells containing Lumogen F Red and the host NLC (Fig. 2a,c). We imaged the sample using epifluorescence microscopy after each centrifugation step to ensure that the shells remained undamaged. To quantify the stability of encapsulation in NAMs, we measured the Lumogen F Red fluorescence intensity, as a proxy for the dye concentration in the NAMs, over 5 months. At each time, we deposited the NAMs onto a glass slide, overlaid the slide with a cover slip, and sealed the edges with wax to minimize changes in the sample due to the diffusion of oxygen and water. Visually, we observed a small decrease in the emission with time, ~20% over 150 days (Fig. 2a–c), and the spatially integrated fluorescence intensity exhibited linear quenching (Fig. 2d). Compared with most dyes, Lumogen F Red is a highly stable emitter, although it does display a small degree of photo bleaching^46^. A control with Lumogen F Red dispersed in NLC and imaged under identical conditions for 10 days demonstrated that photobleaching is a small but significant factor in quantifying the evolution of intensity of Lumogen F Red in our experiments (Fig. 2d, inset). In fact, after 10 days, encapsulation in the NAM appeared to reduce the effects of photo bleaching compared to the dye in the bulk NLC in our control. We thus conclude that Lumogen F Red remains stably encapsulated in the NAM for at least 5 months with minimal to no leakage, testifying to the high containment capability of our structures.

### Plasmon actuation

**Figure 3:**
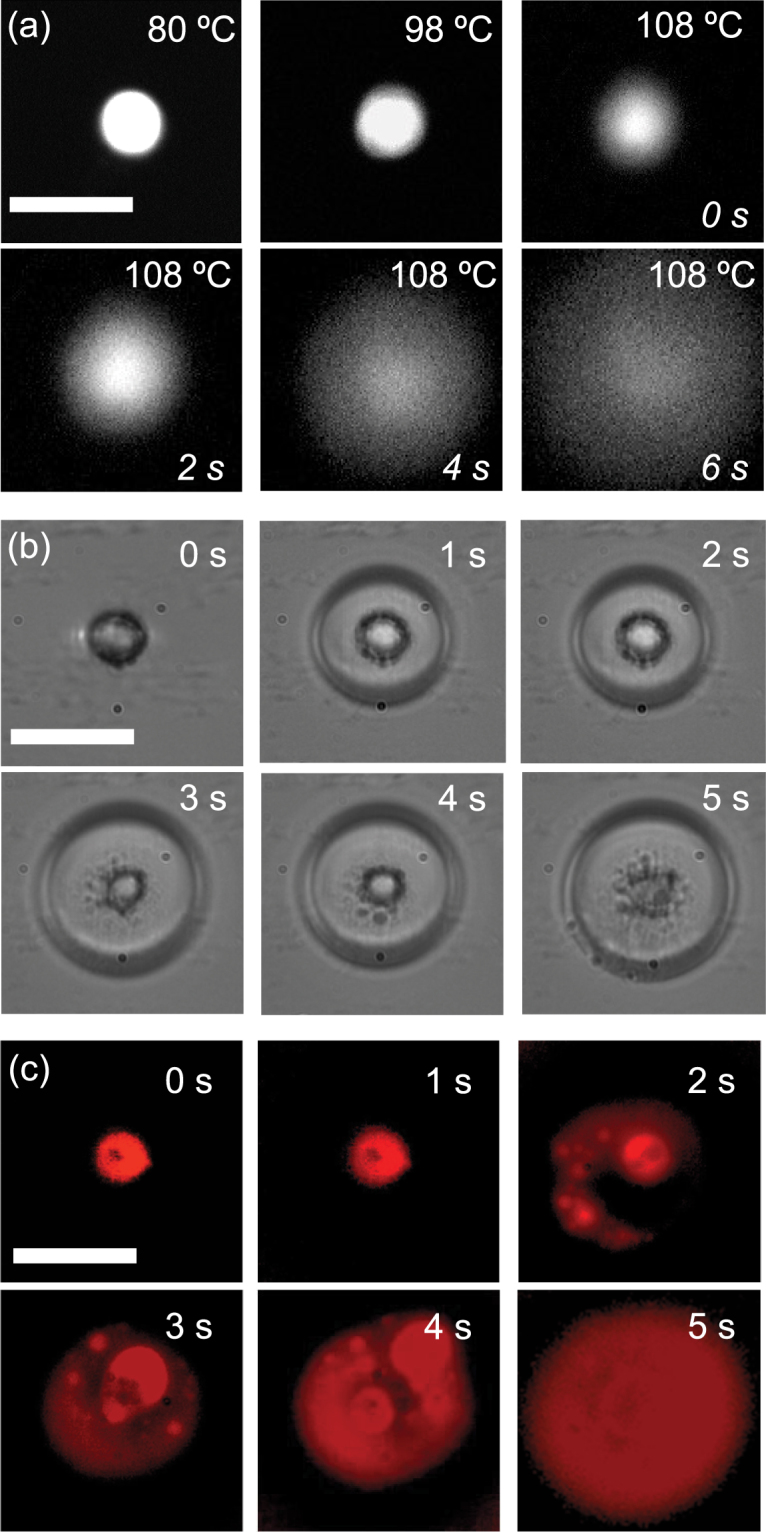
Actuation leading to release of contents from a NAM. (**a**) Fluorescence microscopy images of a NAM loaded with a fluorescent dye on a temperature-controlled stage. The temperature was increased from 80 to 108 °C, and the time after reaching 108 °C is given in the lower right corner. (**b**) Bright-field and (**c**) fluorescence time-lapse images during plasmon-actuated shell disintegration. The encapsulated dye is released during 5 s of illumination with 2 mW of incident power. Scale bars: 3 μm.

**Figure 4:**
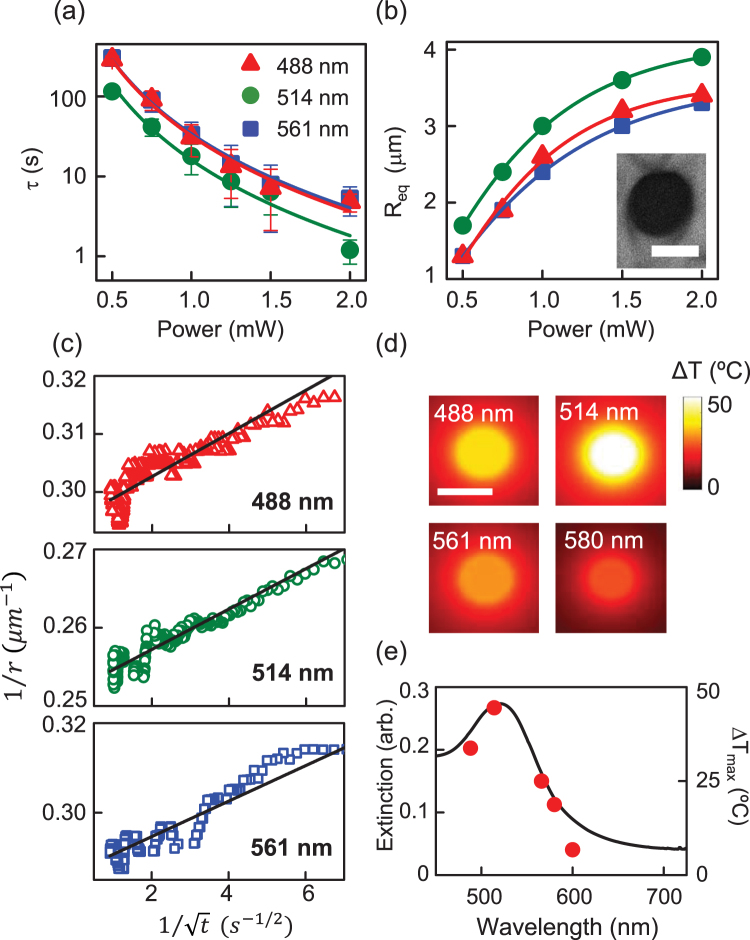
Spectral dependence of photothermal bubble formation. (**a**) The release time *τ* decreases with increasing power for three different excitation wavelengths; the fastest release is achieved at 514 nm, which is the wavelength closest to the LSPR (520 nm). (**b**) The equilibrium bubble radius *R*_eq_ increases with increasing power, and is largest at 514 nm. (inset) cross-polarized image of the bubble shows isotropic phase inside and nematic phase outside. Scale bar: 3 μm. (**c**) The bubble radius *r*(*t*) increases over the first 100 ms of excitation at each wavelength. (**d**) Simulated thermal maps over a range of excitation wavelengths showing that photothermal temperature changes remain strongly localized to the NAM surface. Scale bar: 1 μm. (**e**) The extinction spectrum of a NAM with resonance at 520 nm (curve) shows good agreement with the maximum temperature change at the shell surface (filled circles).

To explore the thermal stability of the shells, we monitored the fluorescence of a shell containing Lumogen F Red while the sample was heated on a temperature-controlled Linkam stage at 1 °C/min, beginning at 25 °C, shown in Fig. 3a. The shell structure remained unchanged up to a temperature of 98 °C. Note that the NLC transitions to its isotropic phase at 35.5 °C, demonstrating that the shells, once assembled, can survive transitions of the host medium. At 98 °C, the shell began to deform slightly. At 108 °C, the rigid shell disintegrated and the dye leaked out, with the structure completely collapsing within 6 s. Differential scanning calorimetry (DSC) studies confirmed that the mesogenic molecules exhibit a crystalline-to-nematic transition around 76 °C and a transition to isotropic at ~132 °C (Figure S3). We propose that this massive structural change of the shell was likely caused by weakened interactions between the ligand molecules as the ligands transitioned to the isotropic state. The lower apparent transition temperature (compared to the 132 °C measured using DSC) can be attributed to the presence of AuNPs. Having confirmed that the NAMs can be destroyed through thermal perturbations and their enclosed cargo released, we sought to trigger release though LSPR-mediated stimuli. We illuminated the NAMs with a 488-nm laser at a power of 2 mW at room temperature, and acquired a series of bright-field images at an interval of 5 s to monitor the structure of the shell (Fig. 3b). After approximately 3 s of illumination, the shell began to disintegrate, and by 5 s it had collapsed completely. Simultaneous fluorescence imaging confirmed that the encapsulated dye leaked out within 2 s of illumination (Fig. 3c), and was released entirely after 5 s. We characterized the release time, τ, as a function of incident power for different excitation wavelengths (Fig. 4a). As expected, we observed the fastest response for excitation at 514 nm (Figure S4), which is closest to the LSPR (520 nm). For the highest incident power (2 mW), release was extremely rapid (τ = 1.2 s). More importantly, release within 2 min was achieved with power as low as 0.5 mW. Furthermore, the NAMs responded to slightly off-resonant light (488 and 561 nm) almost as well, with τ ranging from ~6 s to 5 min with varying incident power (Figure S5), increasing the responsive spectral band to ±40 nm around the LSPR. Tuning the excitation wavelength farther away failed to elicit any response even at higher powers; this spectral selectivity is attractive, as it allows targeting of specific NAMs based on the size of AuNPs constituting the shell wall.

### Modeling the photothermal heating

We noted in our time-lapse imaging of shell disintegration (Fig. 3b) that after 1 s a bubble had formed around the shell. Bubble formation around metallic NPs due to optical excitation is a consequence of the photothermal effect, caused by the local temperature increase inducing a phase transition of the surrounding host medium^47–50^. AuNPs suspended in water are known to produce steam under laser irradiation, and even with sunlight^51^. In our case, POM images confirmed that the temperature increase on and near the shell surface drives a transition in the surrounding NLC from the nematic to isotropic phase, which is what constitutes the bubble (Fig. 4b, inset). The NLC outside remains in the nematic phase, with the bubble radius demarcating the phase boundary. We sought to use the dynamics of the expanding phase boundary as it approached equilibrium to infer the heat flux that propagates through the surrounding medium^50,52^, and more importantly, estimate the resultant increase in temperature. High temperatures from the photothermal effect would suggest that the plasmon-actuated mechanism could result in damage to thermally-sensitive encapsulants or biological materials, nullifying any other benefits the platform could provide. We start by considering the conduction of heat through the liquid crystalline medium. The primary heat source is supplied by the laser incident on the NAM and is assumed to only induce plasmonic heating without affecting the surrounding liquid crystal directly. The time-dependent temperature distribution around the shell is then determined by the solution to the heat equation. For a spherical heat source of fixed radius and strength (${\dot{Q}}_{in}$), the explicit time dependent solution for the temperature as a function of radial distance from the center of the shell, *T*(*r*), can be expressed in terms of error functions^53^, which, for late times, can written to leading order in the reciprocal of time *t* as:

1 $$\frac{4\pi {\kappa }_{{\rm{LC}}}(T(r)-T(\infty ))}{{\dot{Q}}_{in}}=\frac{1}{r}-\frac{1}{\sqrt{\pi \alpha t}}$$

with α = *κ*_LC_/*ρ*_*V*_ *C*_*P*_, where *κ*_LC_ is the thermal permeability of the liquid crystal, *ρ*_*V*_ is the LC density, *C*_*P*_ is the specific heat, and *T*(∞) is the ambient temperature far from the shell. Extracting ${\dot{{Q}}}_{{in}}$ is critical for estimating the temperature distribution near the NAM, as there is no direct route of measuring the heat supplied to each shell. Note that the steady-state solution at infinite time is given by:

2 $$T(r)=T(\infty )+{\dot{{Q}}}_{{in}}/4\pi {\kappa }_{{\rm{LC}}}{r}$$

For simplicity, we ignore the small amount of latent heat associated with the phase change. The location of the phase boundary at infinite time, i.e. the equilibrium bubble radius *R*_*eq*_ is determined by the value of r at which *T*(*r*) is at the nematic-isotropic critical temperature *T*_*NI*_, and is therefore given by

3 $${{R}}_{{eq}}=\frac{{\dot{{Q}}}_{{in}}}{4\pi {\kappa }_{{\rm{LC}}}[{{T}}_{\mathrm{NI}}-{T}(\infty )]}$$

The location of the phase boundary as a function of time is then simply determined by

4 $$\frac{1}{{r}}=\frac{1}{{{R}}_{\mathrm{eq}}}+\frac{1}{\sqrt{(\pi \alpha {t})}}$$

We compared the model above to our experimental data acquired at the highest excitation powers of the expansion of the bubble radius *r*(*t*) (over the first 100 ms of excitation at 488, 514, and 561 nm (Fig. 4c) by fitting the data to Eq. 4. These fits led to an estimate of ${\dot{{Q}}}_{{in}}$, *R*_*eq*_, and *α* using Eq. 2, and we compared these to experimental values for validation. An estimate of *α* using relevant parameters^47,54^ is 1.3 × 10^5^ m^2^/s, while the fits return *α* in the range 0.5–0.9 × 10^5^ m^2^/s. The fitted values of *R*_*eq*_ are 3.5 μm (488 nm), 4 μm (514 nm) and 3.4 μm (561 nm), which compare very well to our experimental *R*_*eq*_ values at 2 mW of incident power (Fig. 4b). From the Eq. 3 above, our calculated ${\dot{{Q}}}_{{in}}$ values are in the range 5–6 × 10^7^ W. As an alternate and independent measurement of the heat generated at the NAM surface, we computed the scattered electric field in the near-field regime of AuNPs in a shell wall, and examined the spatial temperature distribution. The shell was simulated by a random distribution of AuNPs in a spherical arrangement with an average distance of 12 nm, with shell thickness ~160 nm. Using FDTD and discrete dipole scattering (nanoDDSCAT) software^55^, we found that the electric field was consistent with the superposition of fields from the AuNPs, indicating that the AuNPs do not exhibit any inter-particle coupling. We then used the simulated electric field, in combination with measurements that showed typical absorption by a NAM to be ~2–5% of the incident power and a depth of field of ~400 nm, to calculate the heat from a single NAM to be ${\dot{{Q}}}_{{in}}$ = 9 × 10^7^ W, in excellent agreement with the calculated Qin derived from bubble expansion data.

### Accurate thermal mapping

Using Eq. 2, we then computed the spatial dependence of $\Delta {T}\equiv {T}({r})-{T}(\infty )$summed over all AuNPs in a shell for four excitation wavelengths (Fig. 4d). Since heat generation is directly related to the mobility of electrons in the AuNPs, Δ*T* is predictably maximized at excitations tuned to the plasmon resonance frequency. These maps indicate strong localization of photothermal heating, with Δ*T* dropping to half its maximum value within 20–25% of the shell diameter. We determined that the maximum temperature change arising from the photothermal effect is Δ*T*_max_ = 50 °C at the shell surface for excitation at 514 nm near the LSPR, at the highest excitation powers used. Since the samples were maintained at 25 °C, the actual temperature is 75 °C, very close to the crystal-to-nematic transition temperature of the mesogenic ligands. Δ*T*_max_ at different wavelengths showed good agreement with the shell extinction spectrum (Fig. 4e). For biological applications, the heat shock response should enable most bacterial species to survive at higher temperatures during the short time interval required for shell disintegration^56^.

## Conclusion

The success of a drug delivery platform depends on a variety of properties. Our nano-assembled shells provide dramatic improvements on several fronts relative to currently available systems. After encapsulation, there was no significant leakage for over five months, allowing for long-term storage. The optical intensity required for shell disintegration was much lower than that used by other methods, which we attribute to the optimal packing of AuNPs in the shell walls. Furthermore, the time required to release the contents was on the order of seconds, as fast as or faster than other methods. Two aspects of the capsules that would need to be further optimized in the future for *in vivo* applications are size reduction^57^, and actuation by near-infrared (NIR) excitation^58^. With respect to the first, the NAM size distribution is strongly influenced by the rate of cooling the LC host, with faster rates producing smaller shells. We have achieved shell structures with diameters of a few hundred nanometers, and further optimization of this process may lead to further decreases in shell size. The plasmon actuation demonstrated here is not limited by the shell diameter, although it is size dependent. The temperature at the shell surface scales linearly with both the radius and the incident excitation power, which implies that smaller shells will require a higher excitation power to reach the temperature necessary to rupture the structure. This necessity can be mitigated by redesigning the mesogenic ligand. The temperature at which shell structure is disrupted is determined by the liquid crystalline phase transitions of the ligand molecules, and therefore, structural alterations in the mesogenic portion of the ligands will allow us to tune the plasmon actuation of the shells. Because the ligand is easily tailored (see added synthetic scheme, Figure S7), we could increase ratio of aliphatic (sp3) to aromatic C–C bonds to minimize ordering of the ligands in the shells, which could be expected to lower the temperature at which they rupture. Another approach is to shorten the flexible portion of the ligands to allow for higher density of AuNPs, which would also compensate for the excitation power changes. The second, tuning the LSPR to longer wavelengths, is a more complex task^58,59^, and the most well suited Au nanostructures would be hollow nanoparticles where the resonance is shifted by altering shell diameter and thickness^60^. This functionality would add considerable versatility to utilizing these shells *in vivo*, since NIR radiation is capable of deeper tissue penetration compared to visible wavelengths.

The versatility of the shell construction process, in addition to the fact that it is a solution-based one-step synthesis with high yield, allow incorporating these variations relatively straightforward. Coupled with the accuracy of our calculations that allow us to map the temperature changes with high precision, our self-assembled plasmon actuated capsules provide a transformative platform for cargo delivery systems.

## Methods

### Ligand synthesis

Our organic ligand exhibits thermotropic liquid crystalline behavior. When heated, it transitions from a smectic to nematic, and then from a nematic to isotropic phase, as observed in Figure S3. Our functionalized ligands are designed to possess both rigid and flexible segments. The rigid segment, which contains a mesogenic group, contains a relatively rigid rod-like structure. The flexible segment is composed of a long alkyl chain with or without an amine group; the presence of an amine substituent on the chain allows the ligand to attach to the surface of gold nanoparticles. Hence, the flexible segment provides free rotation and fluidity that encourages a close packing of NPs, as well as structural stabilization of our NAMs (Figure S1). The synthesis of the ligand (**S8**) is modeled on prior publications^43,44^ with improvements that are explained in detail in the Supporting Information. The longest linear synthetic sequence consists of five steps (Figure S6).

### Microscopy and spectroscopic methods

Leica DM2500P microscope in the transmission mode with a Leica 63x (NA = 0.80) objective and a Q-image Retiga camera is mounted on the microscope. Polarized optical microscopy and Fluorescence microscopy (reflection) are also carried out on the same microscope. For fluorescence imaging of encapsulated dye, Lumogen F Red (BASF) with a peak emission at 613 nm, we use a 515–560 nm band-pass filter with white-light mercury lamp illumination. Emission was detected using a 580 nm dichroic mirror and a 590 nm long pass filter. Leica DM2500P microscope is used for bright field, cross-polarized, fluorescence images of the NAMs.

Zeiss Gemini SEM 500 instrument operating at 3 kV and 10 kV was used to validate the size of individual NAMs or arrangement of the individual AuNPs in the shell wall. NAMs are extracted from the liquid crystal host and re-dispersed in chloroform after we formed shells in cooling the Liquid crystal-functionalized gold nanoparticle mixture from isotropic to nematic as described earlier. Extracted NAMs were pipetted on Indium Tin Oxide (ITO) glass slides and dried the sample at 80 °C environment for 5 hours. The NAMs are imaged after chloroform is completely evaporated.

Absorption spectral measurements for gold nanoparticles in chloroform are performed using a PerkinElmer UV/Vis spectrophotometer.

### NAMs preparation

The mesogen-functionalized AuNPs (1.2% (wt)) were dispersed into nematic liquid crystal (NLC) 4-Cyano-4′-pentylbiphenyl (5CB, Sigma Aldrich), which exhibits a nematic-to-isotropic transition at 35.5 °C. The NLC-AuNPs mixture was then sonicated in a 40 °C bath for 5 h in an Eppendorf tube, allowing the NLC to remain in the isotropic phase while the solvent (chloroform) in which the AuNPs were suspended during functionalization gradually evaporated from the NLC. Solvent removal was verified by measuring the nematic-to-isotropic phase-transition point using a Perkin-Elmer differential scanning calorimeter (DSC). We place a microscope LC-AuNP mixture slide on a 40 °C maintained Linkham LTS350 hot-stage and verify the sample slide condition that has a great initial dispersion by using a Leica DM2500P upright microscope equipped with a Q-image Retiga camera. After achieving a homogenous dispersion, the mixture was deposited onto a microscope slide mounted on a temperature-controlled Linkam LTS350 hot-stage and sandwiched with a cover slip. All slide and cover clip surfaces were pre-treated with cetyltrimetylammonium bromide (CTAB) or polyvinyl alcohol (PVA) to encourage homeotropic or planar alignment of the NLC molecules. As the NLC-AuNP mixture was cooled into the nematic phase (cooling rate 0.5 °C/s), it separated into NLC-AuNP-rich droplets and an NLC-rich phase. During this segregation, the functionalized AuNPs moved into the shrinking isotropic domains and shell formation was observed. Shell size is controlled by the cooling rate. Fast cooling (3 °C/s) resulted in the formation of shells with diameter ~1 μm, while a slower rate (0.2 °C/s) produced shells as large as 5 μm.

### Encapsulation of fluorescent dye

Lumogen F Red 300 (BASF), dissolved in toluene, is then added to the LC-AuNPs (1.2 wt%) mixture to create concentrations of 2 mM composites. These mixtures are transferred into 1.5 ml Eppendorf tube and sonicated in the 40 °C bath for five hours. After verified complete evaporation of chloroform and toluene by using a DSC, we formed NAMs in the Eppendorf tube by transpiring the tube into a 30 °C maintained modular heating block (VWR). After NAMs are formed, the Eppendorf tube is centrifuged (8000 rpm for 30 min.) to concentrate at the bottom of the tube and the supernatant (LC-Lumogen F Red mixture) is discarded. Add 5CB and mix with the precipitated NAMs in the Eppendorf tube. Repeat this process for 5 times before we image encapsulated dye in the NAMs.

### Thermal stability experiment

Images of NAMs are carried out on Leica DM2500P microscope in transmission and fluorescent mode with a 63x (NA = 0.80) objective. Samples are placed on Linkham LTS350 hot-stage and are heated above the liquid crystal isotropic point (≈35.5 °C) the ligand-ligand interaction continues to maintain a rigid spherical structure on slow heating (1 °C min^−1^).

### Disintegration and bubble expansion images of NAMs

Disintegration and bubble expansion images of NAMs are performed on a TIRF microscope built with a Ti-E Eclipse stand (Nikon Instruments). The objective used is an Apo TIRF 100X (N.A. 1.49). CUBE diode 405 nm laser (Coherent), Sapphire OPSL 488 nm, 514 nm, and 561 nm lasers (Coherent), and OBIS 647 nm (Coherent) are combined into a fiber optic cable and into a TIRF illuminator (Nikon) attached to the microscope stand. Shuttering of the laser illumination is controlled by an acousto-optic tunable filter (AA Optoelectronics) before the fiber coupler. Disintegration images are acquired with an iXon3 + 887 EMCCD (Andor Technology) camera and bubble expansion images are taken by a high-speed camera (Fastec Imaging). Synchronization between these components was achieved using μManager^61^ with a microcontroller (Arduino). We image the disintegration and bubble expansion of the NAMs by exposing sample slides to each activation (405, 488, 514, 561, 647 nm) with incident laser power at 0.5, 0.75, 1, 1.5, and 2 mW in a temperature controlled chamber (HaisonTech) at 25 °C. For bubble expansion images, the time and information of the bubble size are exported to Morphometric and Matlab program files for father data analysis and plotting purpose.

### Small angle X-ray scattering method

Small angle X-ray scattering (SAXS) measurements are carried out at the Stanford Synchrotron Radiation Laboratory, beamline 4–2. Liquid crystal-NAMs mixtures were prepared 24 hours before measurements were taken. The sample mixtures are transferred into 1.5 mm quartz X-ray capillaries at the beamline. The capillaries were mounted in transmission configuration using a custom chamber. Measurements were carried at 11 keV for 1 s per exposure. Diffraction patterns are recorded on a CCD detector, then plotted as integrated intensity as a function of q, the scattering vector using the custom Sas Tool analysis software at the beamline.

### Thermal map simulation

The temperature of NAMs was analytically calculated using Matlab computing software. Nanoparticles were randomly arranged in a sphere with an average distance of 12 nm between them and the thickness of the shell being ~160 nm (20%). The total change in temperature due to the ensemble can be calculated by summing ΔT over all the nanoparticles. We use an intensity of 10^7^J/m^2^ for the incoming light assuming an average absorption of 1%, attributed to shielding of the inner layers and other losses.

## Electronic supplementary material

Supplementary figures
