# Supplementary material for: Plasmon-actuated nano-assembled microshells
Source: Sci Rep. 2017 Dec 19;7:17788. doi: 10.1038/s41598-017-17691-6 (PMC5736557; doi:10.1038/s41598-017-17691-6)
Supplement: Supplementary file 1 — Supplementary figures [file 41598_2017_17691_MOESM1_ESM.doc]

**Plasmon-actuated nano-assembled microshells**

***Makiko T. Quint^1^, Som Sarang^1^, David A. Quint^1,2^, Amir Keshavarz^1^, Benjamin J. Stokes^1^, Anand B. Subramaniam^4^, Kerwyn Casey Huang^2,3^, Ajay Gopinathan^1^, Linda. S. Hirst^1^, and Sayantani Ghosh^1,*^***

^1^School of Natural Sciences, University of California, Merced, CA 95344, USA

^2^Department of Bioengineering, Stanford University, Stanford, CA 94305, USA

^3^Department of Microbiology and Immunology, Stanford University School of Medicine, Stanford, CA 94305, USA

^4^School of Engineering, University of California, Merced, CA 95344, USA

^*^Correspondence: [sghosh@ucmerced.edu](mailto:sghosh@ucmerced.edu)

***Supporting Information****.*



**Figure S1. Ligand structure and binding to AuNP**. Schematic of mesogenic ligand attached to AuNP.

**Figure S2. Small angle x-ray scattering.** Filled arrow shows the scattering peak from the functionalized AuNPs while the broader peak at longer scattering vector is from the 5CB molecules. The shorter q value of 0.05 Å−1 corresponds to a separation of 12.1 nm.

**Figure S3. Differential Scanning Calorimetry (DSC).** The mesogenic ligand shows two peaks in the heating cycle. The first peak at 84 °C denotes a crystalline to nematic-like phase transition, while the second peak (circled) at 134 °C is a nematic to isotropic transition.

**Figure S4.** Bright field images of plasmon-actuated optically induced shell disintegration leading to encapsulated dye being released over 1.4 s of illumination at 2 mW of incident power. Excitation wavelength is 514 nm.

**Figure S5.** Bright field images of plasmon-actuated optically induced shell disintegration leading to encapsulated dye being released over 5.5 s of illumination at 2 mW of incident power. Excitation wavelength is 561 nm.

**Figure S6. Synthesis of the mesogenic ligand.** The rod-like arm **S7** and the amine linker **S4** are made from commercial starting materials in one step (see Figure S6 for details). The synthesis starts with Fischer esterification of **S1** to give **S2** in quantitative yield (**S2** is also commercially available). **S3** is prepared by selective Williamson etherification of **S2**. The amine linker **S4** was then attached to **S3** to afford **S5**. Base-mediated hydrolysis of ester **S5** gave carboxylic acid **S6** in excellent yield. Finally, this compound was coupled to the rod-like arm **S7**, and the product **S8** was isolated by silica gel chromatography. Yields in the final step proved unpredictable: the ester and amine functional groups tend to react, leading to decomposition.

**Figure S7.** **All steps involved in the synthesis of the mesogenic ligand.**

**S2**, **S9**, **S10**, **S11**, and **S12** are commercially available from chemical suppliers.

**Methyl 2-hydroxy-4-(octyloxy)benzoate (S3).** ^1^H NMR (400 MHz, CDCl_3_): δ 10.95 (s, 1H), 7.72 (d, *J* = 9.6 Hz, 1H), 6.43 (s, 1H), 6.43–6.40 (m, 1H), 3.96 (t, *J* = 6.7 Hz, 2H), 3.91 (s, 3H), 1.78 (p, *J* = 8.2 Hz, 2H), 1.47–1.39 (m, 2H), 1.35–1.24 (m, 8H), 0.88 (t, *J* = 7.0 Hz, 3H); ^13^C NMR (100 MHz, CDCl_3_): δ 170.4 (CO), 165.2 (C), 163.7 (C), 131.1 (CH), 107.9 (CH), 105.2 (C), 101.1 (CH), 68.3 (CH_2_), 51.9 (CH_3_), 31.8 (CH_2_), 29.3 (CH_2_), 29.2 (CH_2_), 29.0 (CH_2_), 25.9 (CH_2_), 22.6 (CH_2_), 14.1 (CH_3_). ATR-FTIR (thin film): 3207, 2923, 2850, 1674, 1618, 1577, 1444, 1332, 1247, 1181 cm^-1^.

**6-((*tert*-butoxycarbonyl)amino)hexyl methanesulfonate (S4):** ^1^H NMR (400 MHz, CDCl_3_): δ 4.51 (s, 1H), 4.22 (t, *J* = 6.5 Hz, 2H), 3.11 (q, *J* = 6.8 Hz, 2H), 3.00 (s, 3H), 1.75 (p, *J* = 6.5 Hz, 2H), 1.55–1.45 (m, 3H), 1.44 (s, 9H), 1.42–1.31 (m, 3H); ^13^C NMR (100 MHz, CDCl_3_): δ 156.0 (C), 79.1 (C), 69.9 (CH_2_), 40.4 (CH_2_), 37.4 (CH_3_), 29.9 (CH_2_), 29.0 (CH_2_), 28.4 (3CH_3_), 26.2 (CH_2_), 25.1 (CH_2_).

**Methyl 2-((6-((*tert*-butoxycarbonyl)amino)hexyl)oxy)-4-(octyloxy)benzoate (S5)**. The spectral data matched those reported by Hirst and coworkers.^1^ ^1^H NMR (400 MHz, CDCl_3_): δ 7.82 (d, *J* = 8.6 Hz, 1H), 6.68–6.43 (m, 2H), 4.57 (br s, 1H), 3.98 (q, *J* = 6.4 Hz, 4H), 3.84 (s, 3H), 3.16–3.06 (m, 2H), 1.89–1.72 (m, 4H), 1.53–1.45 (m, 5H), 1.43 (s, 9H), 1.37–1.22 (m, 11H), 0.88 (t, *J* = 7.0, 3H); ^13^C NMR (100 MHz, CDCl_3_): δ 166.3 (C), 163.7 (C), 160.8 (C), 156.0 (C), 133.8 (CH), 112.2 (C), 105.1 (CH), 100.3 (CH), 77.2 (C), 68.7 (CH_2_), 68.2 (CH_2_), 51.6 (CH_3_), 40.5 (CH_2_), 31.8 (CH_2_), 29.3 (CH_2_), 29.2 (CH_2_), 29.1 (CH_2_), 29.0 (CH_2_), 28.8 (CH_2_), 28.4 (3CH_3_), 26.4 (CH_2_), 26.0 (CH_2_), 25.7 (CH_2_), 22.6 (CH_2_), 14.1 (CH_3_). ATR-FTIR (neat): 3375, 2927, 2856, 1704, 1608, 1506, 1250, 1175 cm^-1^.

**2-((6-((*tert*-butoxycarbonyl)amino)hexyl)oxy)-4-(octyloxy)benzoic acid (S6):** ^1^H NMR (400 MHz, CDCl_3_): δ 10.72 (br s, 1H), 8.10 (d, *J* = 8.7 Hz, 1H), 6.61 (dd, *J* = 8.8, 2.3 Hz, 1H), 6.48 (d, *J* = 2.3 Hz, 1H), 4.53 (br s, 1H), 4.19 (t, *J* = 6.7 Hz, 2H), 4.00 (t, *J* = 6.7 Hz, 2H), 3.16–3.07 (m, 2H), 1.91 (p, *J* = 7.8 Hz, 2H), 1.79 (p, *J* = 8.2 Hz, 2H), 1.56–1.45 (m, 5H), 1.43 (s, 9H), 1.42–1.24 (m, 11H), 0.88 (t, *J* = 6.6, 3H); ^13^C NMR (100 MHz, CDCl_3_): δ 165.3 (C), 164.6 (C), 158.9 (C), 156.0 (C), 135.4 (CH), 110.2 (C), 107.1 (CH), 99.8 (CH), 79.1 (C), 70.0 (CH_2_), 68.6 (CH_2_), 40.3 (CH_2_), 31.8 (CH_2_), 29.9 (CH_2_), 29.3 (CH_2_), 29.2 (CH_2_), 29.0 (CH_2_), 28.8 (CH_2_), 28.4 (3CH_3_), 26.3 (CH_2_), 25.9 (CH_2_), 25.6 (CH_2_), 22.6 (CH_2_), 14.1 (CH_3_). ATR-FTIR (neat): 3303, 2927, 1608, 1533, 1439, 1267, 1197, 1126 cm^-1^. HRMS (ESI) *m*/*z* calculated for C_26_H_43_NO_6_ [M]^+^: 466.3163, found: 466.3117.

**4'-Hydroxy-[1,1'-biphenyl]-4-yl 4-(octyloxy)benzoate (S7):** ^1^H NMR (400 MHz, CDCl_3_): δ 8.16 (d, *J* = 8.8 Hz, 2H), 7.56 (d, *J* = 8.7 Hz, 2H), 7.45 (d, *J* = 8.7 Hz, 2H), 7.24 (d, *J* = 8.7 Hz, 2H), 6.98 (d, *J* = 8.7 Hz, 2H), 6.89 (d, *J* = 8.7 Hz, 2H), 4.05 (t, *J* = 6.8 Hz, 2H), 1.83 (p, *J* = 6.8 Hz, 2H), 1.48 (p, *J* = 6.8 Hz, 2H), 1.38–1.26 (m, 8H), 0.89 (t, *J* = 6.7 Hz, 3H): ^13^C NMR (100 MHz, CDCl_3_): δ 165.1 (C), 163.5 (C), 155.1 (C), 150.0 (C), 138.4 (C), 133.2 (C), 132.3 (2CH), 128.4 (2CH), 127.7 (2CH), 122.0 (2CH), 121.5 (C), 115.6 (2CH), 114.3 (2CH), 68.3 (CH_2_), 31.8 (CH_2_), 29.3 (CH_2_), 29.2 (CH_2_), 29.1 (CH_2_), 26.0 (CH_2_), 22.6 (CH_2_), 14.1 (CH_3_). ATR-FTIR (neat): 3458, 2920, 2853, 1748, 1606, 1497, 1254, 1166 cm^-1^.

**4'-((4-(Octyloxy)benzoyl)oxy)-[1,1'-biphenyl]-4-yl 2-((6-aminohexyl)oxy)-4-(octyloxy)benzoate (S8).** ^1^H NMR (400 MHz, CDCl_3_): δ 8.16 (d, *J* = 8.8 Hz, 2H), 8.05 (d, *J* = 9.1 Hz, 1H), 7.61 (d, *J* = 8.6 Hz, 2H), 7.60 (d, *J* = 8.4 Hz, 2H), 7.27 (d, *J* = 8.6 Hz, 2H), 7.26 (d, *J* = 8.4 Hz, 2H), 6.98 (d, *J* = 8.7 Hz, 2H), 6.53 (dd, *J* = 9.0, 6.5 Hz, 1H), 6.49 (d, *J* = 2.5 Hz, 1H), 4.05 (t, *J* = 6.5 Hz, 2H), 4.02 (t, *J* = 6.5 Hz, 2H), 4.01 (t, *J* = 6.5 Hz, 2H), 2.81 (br s, 2H), 2.67 (t, *J* = 6.4 Hz, 2H), 1.91–1.77 (m, 6H), 1.57–1.43 (m, 9H), 1.41–1.26 (m, 17H), 0.91 (t, *J* = 6.5 Hz, 3H), 0.90 (t, *J* = 6.5 Hz, 3H); ^13^C NMR (125 MHz, CDCl_3_): δ 167.9 (C), 167.3 (C), 166.8 (C), 166.3 (C), 164.5 (C), 153.2 (C), 153.1 (C), 140.7 (C), 140.3 (C), 137.1 (CH), 135.0 (2CH), 130.9 (4CH), 125.0 (2CH), 124.8 (2CH), 124.0 (C), 117.0 (2CH), 113.3 (C), 108.2 (CH), 102.8 (CH), 71.4 (CH_2_), 71.0 (2CH_2_), 42.6 (CH_2_), 34.5 (CH_2_), 32.0 (2CH_2_), 31.9 (2CH_2_), 31.8 (CH_2_), 31.7 (CH_2_), 31.3 (CH_2_), 29.7 (CH_2_), 28.7 (2CH_2_), 28.6 (CH_2_), 28.0 (CH_2_), 26.8 (CH_2_), 25.3 (2CH_2_), 16.8 (2CH_3_). ATR-FTIR (neat): 2923, 2854, 1726, 1605, 1251, 1196, 1162 cm^-1^. HRMS (ESI) *m*/*z* calculated for C_48_H_63_NO_7_ [M]^+^: 766.4677, found: 766.4659.
